# Non-instrumental information seeking is resistant to acute stress

**DOI:** 10.1038/s41598-023-46766-w

**Published:** 2023-11-09

**Authors:** Stefan Bode, Matthew Jiwa, Chelsea Chum, Leilani Frost, Hauke R. Heekeren, Katja Wingenfeld, Christian E. Deuter

**Affiliations:** 1 Melbourne School of Psychological Sciences, The University of Melbourne, Melbourne, 3010 Australia; 2 Department of Education and Psychology, Freie Universität Berlin, 14195 Berlin, Germany; 3 Universität Hamburg, 20148 Hamburg, Germany; 4 Department of Psychiatry and Neurosciences, Charité – Universitätsmedizin Berlin, 12203 Berlin, Germany; 5 DZPG (German Center for Mental Health), 12203 Berlin, Germany

**Keywords:** Psychology, Human behaviour

## Abstract

Previous research has shown that people intrinsically value non-instrumental information, which cannot be used to change the outcome of events, but only provides an early resolution of uncertainty. This is true even for information about rather inconsequential events, such as the outcomes of small lotteries. Here we investigated whether participants’ willingness to pay for non-instrumental information about the outcome of simple coin-flip lotteries with guaranteed winnings was modulated by acute stress. Stress was induced using the Socially Evaluated Cold Pressor Test (SECPT), and information-seeking choices were compared to a warm water control group. Our results neither support the hypothesis that stress decreases information-seeking by directing cognitive resources away from the relevance of the lotteries, nor the opposite hypothesis that stress increases information-seeking by driving anxiety levels up. Instead, we found that despite successful stress induction, as evidenced by increased saliva cortisol levels in the SECPT group, information valuation was remarkably stable. This finding is in line with recent findings that experimentally increased state anxiety did not modulate non-instrumental information seeking. Together, these results suggest that the aversiveness of “not knowing” is a stable cognitive state and not easily modulated by situational context, such as acute stress.

## Introduction

The “desire to know” is a strong driver of human behaviour^1–3^. Naturally, one major reason why we value information is that it is often useful to achieve our goals, in particular to maximise rewards or avoid punishments. For example, monitoring the stock prices provides instrumental information to make better investment decisions, and learning about the profile of a planned hike is instrumental to avoid injury. However, it has been argued that there are also other aspects that make information valuable than its instrumental utility. For example, information can make us feel good, allows us to maintain a positive believe state, reduces anxiety, or makes the world more predictable^3–9^.

To dissociate information from its instrumental utility, and to investigate its intrinsic value more directly, we have developed a type of paradigm in which participants can choose to obtain non-instrumental information about the outcome of a series of predetermined lotteries. This information only immediately reveals how much has been won, but it cannot be used to change the odds. Using variations of this paradigm, we have shown that to obtain this information participants are willing to sacrifice substantial proportions of their potential winnings^10–12^, pay small amounts of money^13^, invest physical effort^14^, and even endure physical pain^15^.

Taken together, research on non-instrumental information-seeking has led to the suggestion that information itself might be valuable, i.e. that information is intrinsically rewarding, beyond its instrumental utility e.g.,^3,16,17^. In support of this interpretation, neural signals for information prediction-errors and the subjective value of information have been found to resemble reward-related signals in monkeys^18–20^ and humans^8,12,14^; for a review see^21^.

It is likely that the reason for why humans value non-instrumental information lies in the purpose that is fulfills. In the case of our lottery tasks, modelling results from several studies strongly suggested that the value of obtaining non-instrumental information is related to the reduction of uncertainty^10,11,14^. The presence of non-instrumental information has been shown to directly increase risk-taking in a gambling task, simply because it reduces uncertainty earlier^22^. Higher anxiety and negative affect, but not the personality traits Openness/Intellect, have been shown to increase the willingness to pay for this information^11,23^. The finding that participants are also willing to pay with pain for non-instrumental information^15^ further suggests that the uncertainty of not knowing might be sufficiently aversive that its termination is sometimes worth accepting a physically aversive state in return. In sum, these findings suggest a strong desire to reduce uncertainty, even though the uncertainty of not knowing the outcome of insignificant lotteries has no real relevance either.

In this study, we asked whether acute stress modulates non-instrumental information-seeking behaviour. This allowed us to test how stable information valuation (and the respective desire to reduce uncertainty) is. It has been shown that under acute stress, cognitive and emotional processing substantially changes, which includes attention, cognitive control, learning, memory, risk-taking, decision-making, reward processing, and goal-directed behaviour^24–32^. In general, stress is causing people to shift to cognitively less-demanding strategies^33,34^. One hypothesis is therefore that acute stress triggers a redirection of recourses away from unnecessarily demanding cognitive processes, which in turn should reduce the importance of the uncertainty of not knowing the outcome of the lotteries. This means, acute stress should reduce the willingness to pay for non-instrumental information. The alternative hypothesis is that, because acute stress is reliably associated with negative affect^35^, and negative affect has been linked to increased information-seeking^11^, the salience of uncertainty, including those of the lottery outcomes, might increase. In consequence, participants should be willing to pay more for non-instrumental information. Contrary to both hypotheses, a recent study has experimentally induced state anxiety and has found that there was no effect on information-seeking behaviour in a task comparable to ours in which participants could receive updates on a stock market portfolio^6^. The final hypothesis is therefore that, despite the link between stress and state anxiety, the desire to reduce uncertainty might not be affected by acute stress. Such a finding would suggest that the aversive experience of not knowing is sufficiently stable and not easily modifiable by the situational context. In this case, there should be no modulation of the willingness to pay for non-instrumental information.

In the laboratory, stress can reliably be induced using the Socially Evaluated Cold Pressor Test (SECPT) in which participants are asked to immerse their hand in a bucket of ice water for several minutes, which induces physical stress. At the same time, they are also closely monitored by the experimenter to induce additional socio-evaluative stress^35,36^. This procedure has been shown to result in changes in multiple hormones, neurotransmitters, and peptides^35^. In particular, the socio-evaluative stress component reliably leads to a boost in the cortisol response, both in men and women, that peaks 25–30 min after stress induction^35^.

In this study, we exposed one group of participants to the SECPT while another group received a non-stressful control treatment, which consisted of holding their hand in pleasantly warm water without social evaluation^35^. Following this, we administered an established and sensitive information-seeking task in which participants observed a series of predetermined coin-flip lotteries^15^. In each trial, both sides of the coin were associated with point values, which were later converted into monetary rewards. The mapping between values and sides of the coin, however, were hidden, meaning that while participants knew that all winnings were always added to their total, the exact amount (i.e. the points) were not revealed immediately. Importantly, because the lotteries were predetermined, and knowing the mapping of values to the sides of the coin could not change the outcome, this information was non-instrumental. Participants could nevertheless bid small amounts of money for learning the outcome in each trial immediately. The amount they were willing to pay served as a direct measure for the subjective value they assigned to obtaining this information^13,15^. As in previous work^15^, we varied the expected value of the lotteries between trials, i.e. how much was at stake on average, as well as the range between values associated with each side of the coin, as these variables have been shown to systematically modulate the perceived importance of the lottery^8^. Our main research aim was to test whether being in a state of acute stress modulated information-seeking behaviour in line with one of the hypotheses outlined above.

## Methods

### Participants

*N* = 71 participants were recruited via flyers and online advertisements and randomly assigned to either the stress condition or the control condition. After excluding four participants for incomplete data, *n* = 34 were retained for the stress condition (M_age_ = 23.12; SD_age_ = 3.82; 21 female, 11 male, 2 other) and *n* = 33 for the control condition (M_age_ = 22.94, SD_age_ = 4.42; 23 female, 10 male, 0 other). Participants provided informed written consent and received AUD 20 reimbursement for their time, in addition to their final winnings minus what they spent bidding for information. (Note that we rounded up the final winnings to AUD 10 for all participants, which was equivalent to, or slightly above, participants’ expectations. Paying this small bonus was not done to deceive participants, who did not know their running total throughout the experiment, but for reasons of equity). The study was conducted in accordance with the Declaration of Helsinki, and study protocols were approved by The University of Melbourne Human Research Ethics Committee (ID 2056377.1).

### Stimuli

Participants were asked to decide in each trial how much money of a small budget (determined by their final winnings) they were willing to bid in an auction to receive non-instrumental information about the outcome of a coinflip lottery. To create the lotteries, amounts between 5 and 95 points were combined and randomly associated with either the red or the blue side of a coin, resulting in five different *Expected Values* (EV) (i.e. the average of both sides of the coin; 30, 40, 50, 60, 70) and five levels of *Range* (i.e. differences between reward amounts: 10, 20, 30, 40, 50). Each of these 25 combinations was randomly selected once in each of the three experimental blocks. The probability of each side winning was *p* = 0.5 in each trial. Participants were explicitly instructed that these points would be exchanged for real money after the experiment, with a maximum of AUD 10 to be won.

### Paradigm

**Figure 1:**
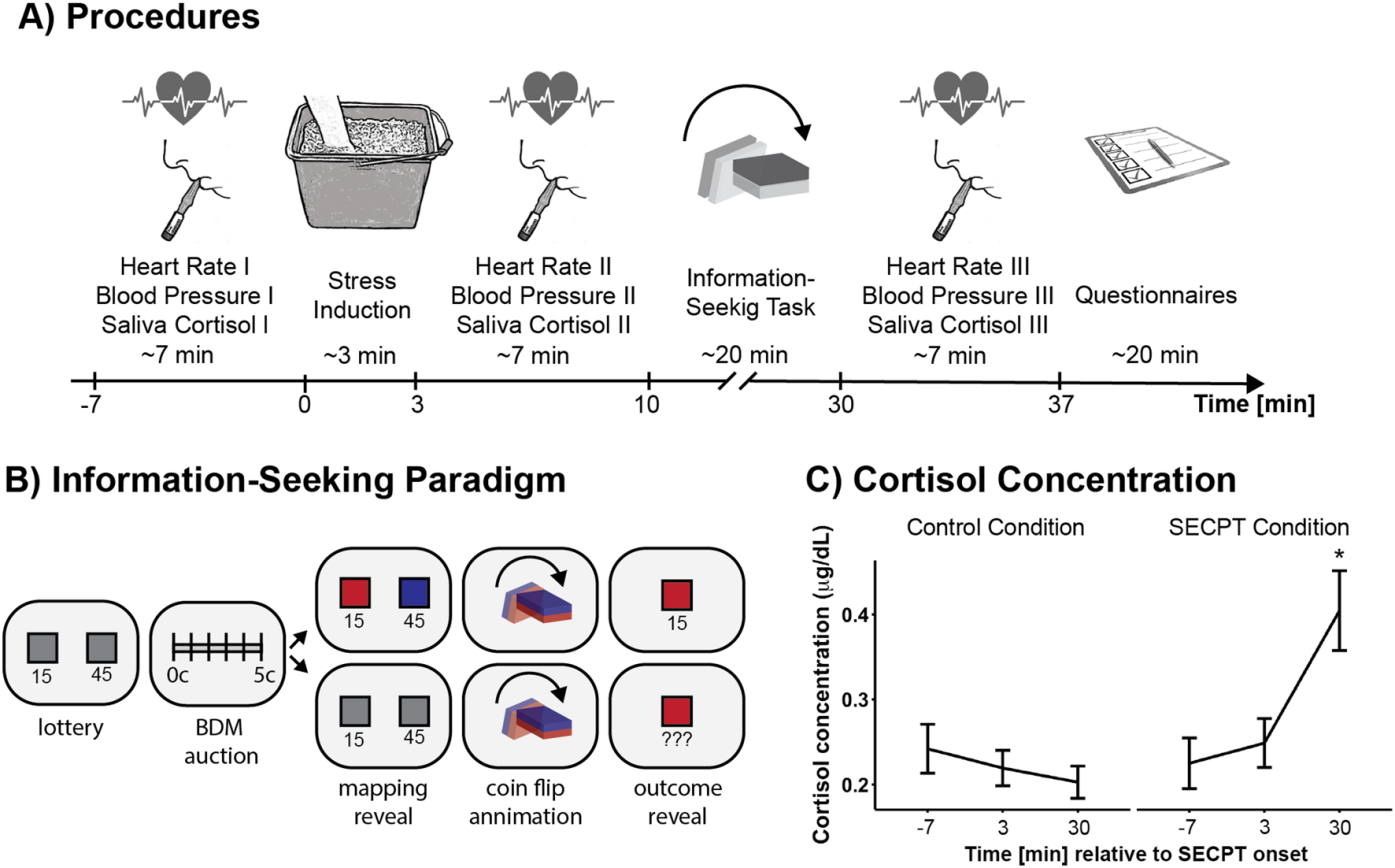
(**A**) Experimental procedures. First, baseline measures of heart rate, blood pressure and cortisol saliva concentration were taken. This was followed by either the SECPT stress induction or the warm water control procedure, after which all physiological measures were taken again. This was followed by the information-seeking task, and subsequently the third measurement of the physiological variables. (**B**) Information-seeking paradigm with auction procedure. In each trial, participants were shown the two amounts that could be won in the coin flip lottery. They then indicated how much (from 0 – 5 cents) they were willing to bid to learn the mapping between colours of the coin and the points (which were later converted to money). If their bid matched or exceeded a random, hidden price set by the computer for this trial, they paid this price, and the mapping was revealed. If their bid was below the hidden price, only the grey sides were shown again. This was followed by an animation of the coin flip. Finally, the outcome colour was displayed, but the final reward outcome was only displayed in case the information was obtained before. However, the winnings were always added to their running total. (**C**) Cortisol concentration for control condition (left) and SECPT condition (right), showing elevated cortisol levels after stress induction at measurement timepoint 3 (post-experiment).

In each trial, participants were shown a screen displaying both sides of the coin, together with the point values that each side would earn them if it won. This means, participants knew the expected value (i.e. the average of both amounts) and the range (the difference between both amounts), but they did not know which colour would earn which amount as both sides were displayed in grey. Below, participants were shown a bar, ranging from 0 (cents) to 5 (cents), and they were instructed to move the slider to the position corresponding to the amount they were willing to bid in exchange for learning the colours associated with the point values for this coin flip. In each trial, the computer set a hidden price for this information, randomly drawn from a uniform distribution. Participants were only shown the information if their bid matched or exceeded this price. In this case, the price was deducted from their budget, and a coin-flip animation was shown, followed by an outcome screen showing the amount won. If the bid was lower than the hidden price, they were also shown the coinflip animation, but followed by a screen showing “???” instead of the amount won as the mapping between amounts and sides of the coin remained hidden (Fig. 1B). The optimal strategy here was to bid exactly the amount that corresponded to the subjective value participants assigned to learning the information. This Becker-DeGroot-Marschak (BDM) auction procedure^37^ has successfully been used in previous work to establish the subjective value assigned to non-instrumental information^13,15^. Importantly, participants were clearly instructed that all amounts won in the lottery would be added to their total winnings, independent of their knowledge of the mapping in each trial. This means that the information was truly non-instrumental, and successfully bidding for the information always reduced their total winnings.

### Procedures

An overview of the procedures can be found in Fig. 1A. Two female experimenters conducted this study, equally often for the SECPT condition and the control condition, with participants (of both sexes) randomly assigned to the conditions.

*Preparation & Training* Upon arrival, participants were pseudo-randomly assigned to either the stress condition or the control condition. They then read the plain language statement and provided written consent to participate in the study. Next, their demographic data was recorded. Participant read the instructions for the information-seeking task, and the experimenter answered questions (while maintaining a reserved expression). The participants then performed several minutes of test trials, until they fully understand the task.

*Baseline physiological measures 1* The experimenter assisted participants with putting on the Polar H10 belt to record their heart rate on a paired Polar V800 watch. After one minute waiting time, a baseline heart rate measure was obtained for 30 s while the participants rested. Following this, a baseline blood pressure measure using an Omron HEM7144T1 cuff blood pressure monitor was obtained. Finally, a baseline saliva sample was obtained using a passive drool tool. The experimenter then labelled the tube with the participant ID and sample ID and stored the sample at − 30 °C in a freezer.

*Stress induction* Depending on their randomisation, participants were subjected to either the Socially Evaluated Cold Pressor Test (SECPT) or the warm water control condition. For the set-up and instruction, we closely followed the procedures outlined by Schwabe and Schächinger^35^. In the SECPT condition, participants were asked to immerse their hand in a bucket with ice water for 3 min. The experimenter informed the participants that they should leave their hand in the water until told by the experimenter to remove it, and that they should only remove it earlier if they felt that they really could not tolerate the ice water any longer. Before the ice water procedure started, the experimenter adjusted a camera mounted onto a tripod, zooming directly in the participants’ faces, with the screen adjusted such that participants could see their own faces. Participants were instructed to look into the video camera. The experimenter stayed in the room for the duration of the procedure, taking notes, with a reserved expression, maintaining only minimal interaction and avoiding any form of reinforcement^35^. After this phase, the experimenter instructed participants to remove the hand from the bucket and dry it with a paper tissue. In the control condition, participants were asked to put their hand in a bucket with pleasantly warm water for 3 min. No camera was present, and the experimenter, while in the room, paid no attention to the participant but engaged in paperwork on the other side of the room.

*Physiological measures 2* While participants were still seated, the experimenter repeated the measurements of heart rate, blood pressure and took another saliva sample to measure cortisol levels.

*Information-seeking task* Immediately after taking the physiological measures, participants were seated comfortably in a chair, approximately 50 cm distance from a HP monitor (1920*1080 resolution; 60 Hz refresh rate) and performed the information-seeking task on a HP computer (Intel Core i7-8700 processor). The experiment was presented via PsychToolbox-3^38^ running on MATLAB R2017b (The Mathworks, Natick, MA). Each combination of *Expected Value* and *Range* was repeated three times, for a total of 75 trials. The order of the trials was randomised, with randomisation repeated until no two consecutive trials had the same *Expected Value* and *Range*. Participants were given a self-timed break between the three blocks, i.e. after every 25 trials. The experiment took ~ 20 min to complete.

*Physiological measures 3* The experimenter then repeated the measures of heart rate, blood pressure and took another saliva sample to measure cortisol levels.

*Questionnaires* Participants reported their demographic data, filled out the new Big Five Inventory (BFI-2) anxiety and emotional volatility questionnaire^39^, the Big Five Aspects Scale (BFAS) volatility and withdrawal scales^40^, the Five-Dimensional Curiosity Scale-Revised (5DCR)^41^, the Intolerance for Uncertainty Scale (IUS)^42^, and the domain-specific risk-taking scale (DOSPERT scale)^43^. Note that given that the sample size was rather small for testing for individual differences, the questionnaires only served to test for potential differences between groups.

As an additional control measure for the stress induction, (which we note was only introduced halfway through the experiment), we collected subjective stress experience ratings using a Likert scale from 1 “I don’t feel stressed at all” to 10 “I feel extremely stressed” for the final n = 18 control condition participants and the final n = 19 SECPT condition participants.

*End of session* At the end of the session, participants were paid via bank transfer. They were debriefed about the purpose of the study.

### Statistical analyses

We conducted analysis of variance (ANOVAs) with each *Expected Value* (5 levels) and *Range* (5 levels) as the repeated measures factor, and *Stress Group* (SECPT condition and control condition) as the between-subjects factor. *Expected Value*, *Range* and *Stress Group* were treated as categorical variables, and bid amount was the dependent variable. First, we aimed to replicate the main effects of *Expected Value* and *Range* on information-seeking choices from previous work^15^. Our primary interest, however, was (a) the potential main effect for *Stress Group*, i.e. whether stress decreased information-seeking behaviour as compared to the control group, and (b) the interaction terms between *Stress Group* and *Expected Value* and *Range*, respectively. These interactions were informative about whether any potential effect of stress on information-seeking was related to the stake of the lottery and the difference between the amounts to be won. In addition, we conducted mixed effects models to confirm the results. For the *Baseline Model*, we included *Expected Value* and *Range* as fixed effects, and as random effects (i.e. slopes) we used *Participant*, *Expected Value* and *Range.* (Note that this Baseline Model provided a better fit than an alternative Baseline Model, which did not include *Expected Value* and *Range* as additional random effects; data not shown). The *Stress Group Model* was identical to the *Baseline Model* but included *Stress Group* as an additional fixed effect. Models were compared using both the Akaike Information Criterion (AIC) and Bayesian Information Criterion (BIC). Finally, we also conducted an analogous Bayesian ANOVA, with the factors: *Stress Group* (2 conditions), *Expected Value* (5 levels) and *Range* (5 levels) in JASP (JASP Team, 2023) and tested directly for the exclusion of specific factors using Bayes Factors (BF).

To verify that the stress induction was successful, we conducted an ANOVA for the saliva cortisol concentration [μg/dL] with the repeated-measures factor *Timepoint* (pre-induction, post-induction, post-experiment) and the between-groups factor *Stress Group* (SECPT condition, control condition)*.* Given the typical time course of cortisol responses, we expected a successful stress response to be reflected by a significant increase in cortisol concentration measured post-experimentally (timepoint 3) in the SECPT condition only^35^. Additional secondary control ANOVAs were conducted for heart rate, systolic blood pressure, and diastolic blood pressure, again with the repeated-measures factor *Timepoint* and the between-groups factor *Stress Group.*

Finally, we used mixed effects models to test whether in the SECPT condition only participants’ individual saliva cortisol concentration was related to information seeking-behaviour. This analysis was conducted to capture potential differences in stress responses^35^. For this, we compared two models: Both predicted bid size and included *Expected Value* and *Range* as fixed effects, as well as additional random effects for *Participant*, *Expected Value* and *Range*. The *Cortisol Stress Model* included the change in cortisol concentration between the first (pre-induction) and last (post-experiment) samples as an additional fixed effect, while the *Baseline Stress Model* (identical to the *Baseline Model* above, but only fit to the SECPT condition participants) did not. Models were again compared using AIC and BIC.

Note that we did not intend to use the questionnaire measures for statistical analyses as our sample size was much smaller than required for correlational analyses of individual differences (a Pearson correlation of 0.20 at *p* < 0.05 with 80% power requires a sample of N ~ 200). These measures were included for potential future analyses across multiple studies with comparable outcome measures, but some results will be reported here for completeness.

## Results

### Stress induction

All participants completed the full three minutes ice water (and warm water control) procedure. The stress induction was successful, as demonstrated by the significant interaction effect for *Timepoint* * *Stress Group* in the ANOVA for salivia cortisol concentration, F(2, 130) = 15.692, *p* < 0.0001, η2 = 0.068. Cortisol levels were elevated only at timepoint 3 (post-experiment) in the SECPT condition (Fig. 1C). Note that timepoint 2, directly after the stress induction, is too early to show elevated cortisol levels^35^. The cortisol effect measured after the information-seeking task, however, provides direct evidence that the stress induction had a lasting effect while the task was performed. There were no significant *Stress Group* effects nor *Timepoint* * *Stress Group* interaction effects for heart rate, systolic blood pressure, or diastolic blood pressure (see Supplementary Table 1 and 2 for summary statistics and Supplementary Table 3 and 4 for all results). However, in hindsight, we were unlikely to capture any stress-related effects with this protocol as these measures have been reported to peak during the stress induction and return to baseline immediately after^35^, meaning that timepoint 2 was already too late to reflect stress-related effects. A Welch’s t-test was used to compare the subjective stress experience ratings between control group (M = 2.33, SD = 2.40) and SECPT group (M = 6.21, SD = 2.12), revealing significant differences in the stress experience, *t*(33.93) = 5.19, *p* < 0.001, *d* = 1.71.

### Information-seeking

**Figure 2:**
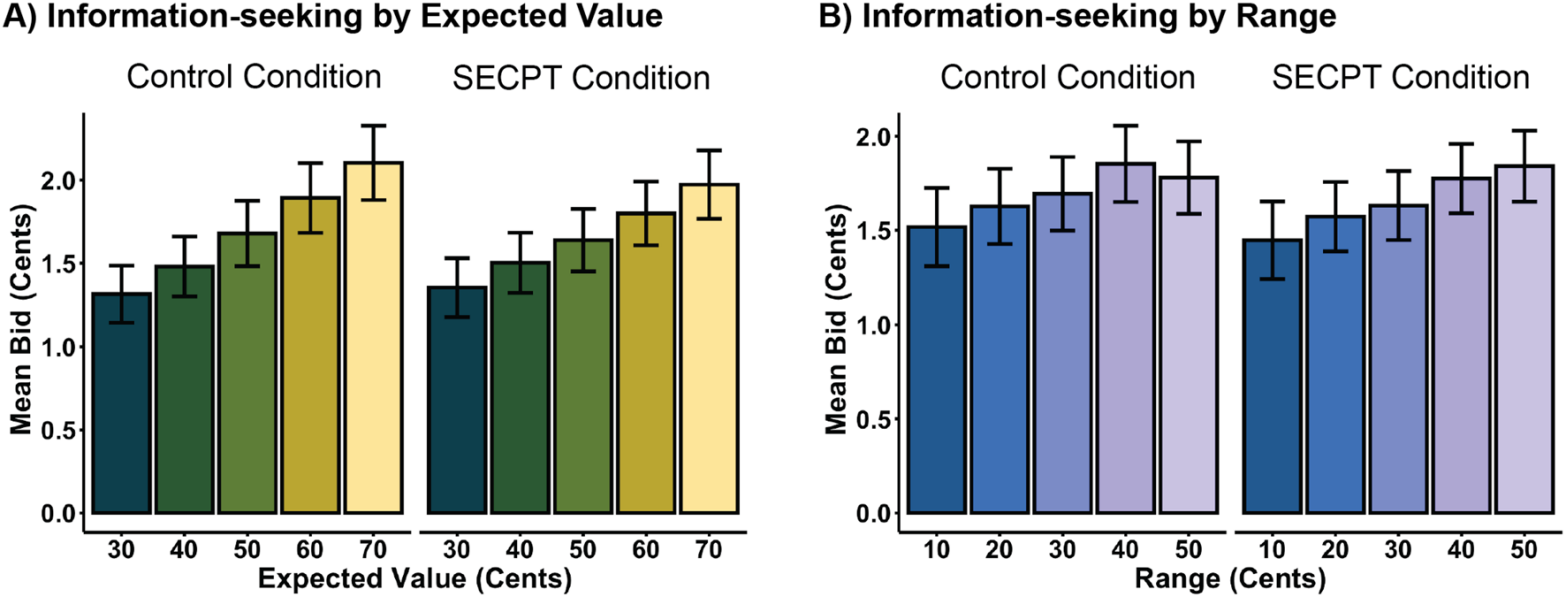
(**A**) Mean bid for non-instrumental information increased as a function of *Expected Value* of the lottery (i.e. the average reward of both sides of the coin). No differences between the control condition (left panel) and the SECPT condition (right panel) were found; (**B**) Mean bid for non-instrumental information increased as a function of *Range* (i.e. difference between reward values of both sides of the coin). No differences between the control condition (left panel) and the SECPT condition (right panel) were found.

Participants were generally willing to pay for non-instrumental information (Fig. 2). The ANOVA results showed a main effect of *Expected Value,* F(4, 260) = 31.876, *p* < 0.0001, η2 = 0.033, and a main effect of *Range*, F(4, 260) = 6.979, *p* =  < 0.0001, η2 = 0.009, replicating pervious findings^15^ that the more was at stake (i.e. the higher the expected value) and the stronger the difference between the two possible rewards (i.e. the higher the range), the more participants were willing to pay for information (Fig. 2). Importantly, the main effect of *Stress Group* was not significant, and there were also no significant interaction effects between *Stress Group* and *Expected Value* or *Stress Group* and *Range* (see Supplementary Table 5 for full results). As an additional validation of these findings, we ran mixed effects models comparing a *Baseline Model* with a *Stress Group Model*, which included the stress condition as an additional fixed effect, and confirmed significant effects for *Expected Value* and *Range*, but no improvement of model fit for the inclusion of *Stress Group*. Finally, we also ran an additional *Stress Group Interaction Model* that included all of the effects in the *Stress Group Model*, as well as fixed interaction effects between *Stress Group* and *Expected Value* and *Stress Group* and *Range*. Neither of these models showed improved fit to the data, indicating that *Stress Group* had neither a main effect nor an interaction effect on participants’ bidding behaviour (see Supplementary Table 6 and 7).

To confirm these results, we further conducted a Bayesian ANOVA, which shows moderate support (using guidelines from^44^) for the exclusion of *Stress Group* (BF = 6.86) and strong support for the exclusion of the interaction terms for *Stress Group* and *Expected Value* (BF = 159.78), *Stress Group* and *Range* (BF = 90.38), as well as for the three-way interaction of *Stress Group*, *Expected Value* and *Range* (BF > 1000). See Table 1 for full results.

**Table 1:** Results of Bayesian ANOVA for Information-seeking.

Effects	P (incl)	P (excl)	P (incl|data)	P (excl|data)	BF_excl_
EV	0.737	0.263	1.000	< .001	< .001
Range	0.737	0.263	0.998	0.002	0.004
EV ✻ Range	0.316	0.684	0.007	0.993	65.262
Stress Group	0.737	0.263	0.290	0.710	6.864
EV * Stress Group	0.316	0.684	0.003	0.997	159.777
Range * Stress Group	0.316	0.684	0.005	0.995	90.381
EV * Range * Stress Group	0.053	0.947	< .001	1.000	> 1000

Note: The table shows the Bayes Factors (BFs) for the exclusion of each factor. EV = Expected Value.

Finally, we analysed whether within the SECPT condition, participants with a larger cortisol response might have showed altered information-seeking behaviour, given that there are well-documented individual differences in the cortisol response to the SECPT^35^. For this, we compared the fit of the *Baseline Stress Model* with the *Cortisol Stress Model*, which included cortisol level as an additional fixed effect. The results showed that including participants’ cortisol concentration as a predictor did not improve the model fit (for full results see Supplementary Table 8).

In summary, our results strongly suggest that information-seeking behaviour was not modulated by the stress induction.

### Questionnaire results

The questionnaire results confirmed that there were no significant differences between the control condition sample and the SECPT condition sample for any of the traits measured by these scales or subscales (all *p* > 0.05; see Supplementary Table 9 for summary statistics, and Supplementary Table 10 for correlations between scales). In an additional explorative analysis step, we correlated the questionnaire scores with individuals’ estimates of model parameters (*Intercept, Expected Value, Range*) from the winning *Baseline Model* (see above). No significant correlations were found after correction for multiple comparisons (see Supplementary Table 11 for all correlations).

## Discussion

In this study, we used an established coin flip lottery task^13,15^ to investigate whether non-instrumental information-seeking behaviour was altered under acute stress. In each trial, participants could bid for receiving information about the mapping of point values to the two sides of the coin, which ultimately provided them with information about the outcome of the lottery; but this information could not be used to change the odds of the lottery, and they always won the amount associated with the winning side. Before performing the task, participants either experienced acute stress induced by the Socially Evaluated Cold Pressor Test (SECPT), which required them to keep their hand in ice-cold water for three minutes while being observed by an experimenter, or they did not experience stress in a control condition, which only required them to keep their hand in pleasantly warm water for the same period. We measured cortisol responses through a series of saliva samples, which showed that the stress induction was successful. Our results demonstrated that there was no modification of information-seeking behaviour in the stress condition. Both the expected value of the lottery as well as the range between available amounts moderated information-seeking; however, these moderation effects did not differ between groups.

Recent work has suggested a strong link between information-seeking for non-instrumental information in similar lottery tasks and the reduction of uncertainty as a potential driver^10,11^. In other words, participants could use the information only to terminate the state of ‘not knowing’, which appears to be intrinsically aversive^15^. We set out to test three different hypotheses about how acute stress might moderate information-seeking behaviour, and there are plausible theoretical reasons for all of them. The first possibility was that stress would reduce non-instrumental information-seeking, because stress might trigger a redirection of cognitive recourses away from unnecessarily demanding cognitive processes. Arguably, reducing the (rather insignificant) uncertainty about the lottery outcomes might be such an unnecessary cognitive process. The second possibility was that stress would increase non-instrumental information-seeking, as stress increases negative affect and anxiety, which in turn might increase the salience of the uncertainty about the lottery outcomes. The third hypothesis was that there would be no modulation of non-instrumental information-seeking, despite an increase in anxiety, which would suggest that the desire to reduce uncertainty is a cognitive state not easily modulated by contextual factors, such as stress. Our result of no modification of information-seeking after the SECPT supports the latter hypothesis. It is also in line with results by Charpentier and colleagues^6^, who have used the Trier Social Stress Test (TSST) with the goal to experimentally increase state anxiety. They have shown that elevated anxiety levels did not increase non-instrumental information-seeking (but found that anxiety was related to increased information-seeking in relation to large changes in the environment). Others have demonstrated that related, decision-relevant cognitive states, such as risk attitudes, loss aversion, and choice consistency, were also not modulated by acute stress^45^, which is consistent with our interpretation that the desire to reduce uncertainty is similarly stable under stress. It should be acknowledged that demonstrating the absence of an effect can, of course, never be fully conclusive. However, our additional mixed effects modelling results, showing that neither including the stress condition as a fixed effect nor including cortisol levels as an additional factor improved the model fit, further supports that there was no stress-related effect. This was also confirmed by a Bayesian ANOVA, with Bayes Factors suggesting moderate support for excluding Stress Group as a factor, and strong support for excluding the interactions between Stress Group and Expected Value as well as Stress Group and Range, respectively.

Our results do not rule out that information-seeking could indeed be modulated by other forms of stress, a prolonged experience of stress, or higher levels of stress intensity. In some of these scenarios, significantly higher levels of state anxiety might be induced, or a stronger redirection of cognitive resources might result, which could potentially modulate information-seeking. However, we are generally confident that the SECPT procedure itself is a valid way to induce stress, as an evaluation of multiple studies showed that the SECPT is reliably experienced as stressful, painful, unpleasant, and difficult^35^. It has also been shown to consistently modify other processes across several cognitive domains, including attention, learning, memory, risk-taking, and goal-directed action^26,28–32^. The elevated levels of cortisol^35^, which were clearly still evident after completion of the information-seeking task, strongly suggest that participants in the SECPT condition were indeed stressed during the information-seeking task. This was confirmed by significant differences in subjective stress ratings between experimental conditions that we acquired for a part of our samples. Crucially, cortisol has been suggested to be the main driver of stress effects on emotion and cognition^26,34,35^. While we did not observe a modulation of heart rate or blood pressure, this was most likely due to our choice of measurement timepoints, as both measures have been reported to be maximal *during* the SECPT procedure and return to baseline quickly directly afterwards^35^.

Testing for potential moderation effects of other types of stress was beyond the scope of this study, and we therefore do not make statements about general stress resistance here. However, our results do suggest that information-seeking related to reducing uncertainty does not easily break down under a standard stress-induction. This, in turn, suggests that the intrinsic value of information in such scenarios is stable, and that short periods of acute stress and anxiety do not have a strong impact on information-seeking behaviour, unless in response to large changes in the environment^6^. It remains to be tested whether there are individual differences in people’s reactions to stress that might interact with their information-seeking behaviour and hence their valuation of information under stress. For example, a fully balanced repeated-measures design could be used to test whether parts of the null effect could be due to some individuals showing opposing effects of stress on information-seeking. Another important question in relation to information-seeking is how closely stress and anxiety are related. Charpentier and colleagues^6^, for example, used a (different but similar) stress induction to evoke state anxiety. The social evaluation component of the SECPT is most likely also triggering anxiety responses as part of the stress response. There is a close link between the cognitive processes and neural mechanisms underlying stress and anxiety^46^, which makes it difficult to dissociate their effects on information-seeking.

A slightly different perspective on our results is that because the experience of uncertainty is an aversive state^15^, it might be this experience that is stable under acute stress. In other words, the value of this information is only a function of how unpleasant uncertainty is, and the individual experience of uncertainty (and the need to reduce it) is more trait-like. We note that our study was not sufficiently powered to investigate this question further. While previous work has failed to produce evidence for a relationship between information-seeking and intolerance for uncertainty^11^, the specific experience of uncertainty related to “not knowing” that is relevant in our study might not have been optimally captured yet.

We further note that our study only investigated one very specific type of information-seeking, which arguably could only reduce a very specific type of uncertainty. Charpentier and colleagues^6^ have shown that the effect of anxiety on information-seeking can be markedly different when this information pertains to more important events. Others have demonstrated that acute stress induced by the SECPT was associated with a higher tolerance of uncertainty, as participants increasingly chose highly variable but larger rewards over stable but smaller rewards^24^. In this previous study, however, uncertainty was not related to the time until its resolution, as in our study, and it was confounded with reward, and no non-instrumental information could be obtained. Finally, in every-day life, people are also strongly driven to actively seek-out other types of non-instrumental information, motivated by curiosity, novelty, or positive affect^2,3,5,47^, which we do not cover here in detail. Our finding that higher expected values were associated with increased information-seeking, however, supports that positive affect is a motivating factor. This effect directly replicates our previous results using the same task^15^ and is also in line with others’ demonstrations of preferences for gaining knowledge about favourable outcomes^5^, and higher bids^13^ and more willingness to invest physical effort^14^ to obtain information when win probabilities in similar random lotteries were higher. Even in a task in which information could be used to make better predictions about lottery outcomes, participants have been shown to prefer learning from sources that made more favourable predictions, over and above being accurate^48^. This confirms that hedonic motives substantially impact information valuation.

There are some other limitations to be considered. It would, for example, be desirable for future research to test a larger sample, which would additionally allow for assessing individual differences, in particular in relation to intolerance to uncertainty^11,49^, clinical disorders related to motivational deficits such as depression and anxiety^50–52^, and sex differences in stress responses^53^. There are also other information-seeking tasks that should be used to replicate the current findings and to understand whether information-seeking behaviour in other situations is equally stable under stress^6,8^. It must further be noted that our concept of “stability” in this study only related to manipulations of one contextual factor, that is acute stress. Another option would be to test whether information-seeking behaviour generally remains stable over time. Finally, we have recently used a variant of the task employed here in which participants could accept painful stimuli instead of money to obtain non-instrumental information^15^. Using pain as an alternative “currency” would also be interesting in this context, because the SECPT also involves experiencing strong discomfort and pain^35^. It would therefore be interesting to understand if pain-related stress could affect participants’ subsequent willingness to accept even more pain to pay for information, either positively through a desensitisation effect, or negatively through a sensitisation effect.

## Conclusions

In summary, this study has replicated previous findings that the expected value and the range of a simple coin flip lottery increased non-instrumental information-seeking. Stress induction via the Socially Evaluated Cold Pressor Test (SECPT), however, did not moderate information-seeking, suggesting that the intrinsic value of information is stable during brief episodes of acute stress. This provides further evidence that the “desire to know”, in particular when related to the experience of uncertainty, is a strong motivational driver and not easily supressed.

### Supplementary Information

Supplementary Information.

## Data Availability

The datasets generated and analysed during the current study are available in the Open Science Framework repository, https://osf.io/v3752/.
