# Supplementary material for: Non-instrumental information seeking is resistant to acute stress
Source: Sci Rep. 2023 Nov 9;13:19505. doi: 10.1038/s41598-023-46766-w (PMC10636112; doi:10.1038/s41598-023-46766-w)
Supplement: Supplementary file 1 — Supplementary Information. [file 41598_2023_46766_MOESM1_ESM.pdf]

Supplementary Materials

Non-instrumental information seeking is resistant to acute stress

Stefan Bode[1*], Matthew Jiwa[1], Chelsea Chum[1], Leilani Frost[1], Hauke R. Heekeren[2,3],

Katja Wingenfeld[4,5], Christian E. Deuter[4]

[1] Melbourne School of Psychological Sciences, The University of Melbourne, Melbourne 3010, Australia

[2] Department of Education and Psychology, Freie Universität Berlin, 14195 Berlin, Germany

[3] Universität Hamburg, 20148 Hamburg, Germany

[4] Department of Psychiatry and Neurosciences, Charité – Universitätsmedizin Berlin, 12203 Berlin, Germany

[5] DZPG (German Center for Mental Health), 12203 Berlin, Germany

* Corresponding author: Stefan Bode, Melbourne School of Psychological Sciences, The University of Melbourne, Melbourne 3010, Australia; email: sbode@unimelb.edu.au

Physiological Variables ANOVA Results

Supplementary Table 1: Summary statistics for physiological data SEPCT group.

	**Timepoint 1 pre-induction**	**Timepoint 2 post-induction**	**Timepoint 3 post-experiment**
Blood cortisol concentration (µg/dL)	0.228 (0.02)	0.247 (0.03)	0.397 (0.40)
Heart rate (bpm)	82.82 (2.218)	79.61 (1.892)	79.70 (1.915)
Systolic blood pressure (mmHg)	114.12 (2.382)	113.70 (2.026)	109.30 (2.673)
Diastolic blood pressure (mmHg)	73.09 (1.47)	74.97 (1.34)	73.00 (1.59)

Note: Shown are means and standard errors.

Supplementary Table 2: Summary statistics for physiological data control group.

	**Timepoint 1 pre-induction**	**Timepoint 2 post-induction**	**Timepoint 3 post-experiment**
Blood cortisol concentration (µg/dL)	0.244 (0.03)	0.222 (0.02)	0.216 (0.02)
Heart rate (bpm)	77.88 (2.424)	77.44 (2.448)	76.94 (2.327)
Systolic blood pressure (mmHg)	112.65 (2.513)	110.71 (2.974)	109.09 (2.468)
Diastolic blood pressure (mmHg)	72.24 (1.463)	73.15 (1.498)	74.24 (1.660)

Note: Shown are means and standard errors.

Supplementary Table 3: ANOVA results for physiological data

	Effect	*F*	*df*	*p*	*η2*
**Blood cortisol concentration**					
	Timepoint	7.682	2, 130	**<.0001**	0.034
	Group	3.892	1, 65	.053	0.04
	Timepoint * Group	15.692	2, 130	**<.0001**	0.068
**Heart rate**					
	Timepoint	6.146	2, 130	.003	0.005
	Group	0.989	1, 65	.324	0.014
	Timepoint * Group	2.592	2, 130	.079	0.002
**Systolic blood pressure**					
	Timepoint	7.689	2, 130	**<.0001**	0.005
	Group	0.143	1, 65	.706	0.002
	Timepoint * Group	0.56	2, 130	.572	0.001
**Diastolic blood pressure**					
	Timepoint	1.483	2, 130	.231	0.005
	Group	0.001	1, 65	.982	0.00001
	Timepoint * Group	1.278	2, 130	.282	0.004

Note: Three Timepoints (pre-induction, post-induction, post-experiment) were used as a repeated-measures factor; Group consisted of the control group and the SECPT group as the between-subject factor. The meaningful interaction Timepoint * Group (indicating a selective change in the stress induction group over time) was significant for blood cortisol.

Supplementary Table 4: Post-hoc *t*-test results for blood cortisol concentration

**Control Group**		
**Contrast**	***t (df)***	***p***
Timepoint 2 – Timepoint 1	1.90 (32)	.067
Timepoint 3 – Timepoint 1	1.75 (32)	.090
Timepoint 3 – Timepoint 2	1.12 (32)	.273
**SECPT Group**		
**Contrast**	***t (df)***	***p***
Timepoint 2 – Timepoint 1	1.46 (33)	.155
Timepoint 3 – Timepoint 1	3.79 (33)	**.0006**
Timepoint 3 – Timepoint 2	3.89 (33)	**.0005**

Note: Paired-samples t-test results. Both significant results for timepoint 3 in SECPT group survive Bonferroni-corrections for multiple comparisons.

Information-Seeking ANOVA Results

Supplementary Table 5: ANOVA results for information-seeking bid size (in cents)

Effect	*F*	*df*	*p*	*η2*
Expected Value	31.876	4, 260	**<.0001**	0.033
Group	0.016	1, 65	.899	<0.0001
Expected Value * Group	0.547	4, 260	.701	<0.0001
Range	6.979	4, 260	**<.0001**	0.009
Group	0.016	1, 65	0.899	<0.0001
Range * Group	0.289	4, 260	0.885	<0.0001

Note: Expected Value = the average reward of both sides of the coin (5 conditions); Range = the difference between reward values of both sides of the coin (5 conditions); Group = SECPT and control group (2 conditions).

Information-Seeking Mixed Effects Modelling Results

Supplementary Table 6: Model comparison predicting information-seeking bid size

Models	AIC	BIC
(1) Baseline Model	**12382**	**12448**
(2) Stress Group Model	12384	12456
(3) Stress Group Interaction Model	12388	12472

Model specifications

Baseline Model:

BID.c ~ 1 + RANGE.c + EV.c + (1 + RANGE.c + EV.c | ID)

Stress Group Model:

BID.c ~ 1 + RANGE.c + EV.c + COND + (1 + RANGE.c + EV.c | ID)

Stress Group Interaction Model:

BID.c ~ 1 + RANGE.c + EV.c + COND + RANGE.c:COND + EV.c:COND + (1 + RANGE.c + EV.c | ID)

The mixed-effects modelling results show that the model that provided the best fit to the data was the *Baseline Model*, which included only *Expected Value* and *Range* as fixed and random effects. Including participants' experimental condition (*Stress Group Model*) and its interactions with *Expected Value* and *Range* (*Stress Group Interaction Model*) did not improve the fit of the model to the data.

Supplementary Table 7: Parameters for *Baseline Model* predicting information-seeking bid size

**Baseline Model Parameters**			
**Predictor**	***ß*** **(SE)**	***t***	***p***
Intercept	0.626 (0.16)	3.927	**<.0001**
Expected Value (EV)	0.249 (0.04)	2.877	**<.0001**
Range	0.123 (0.04)	6.333	**<.0001**

Note: Expected Value (EV), Range were continuous variables. Only statistics for fixed effects are reported.

Supplementary Table 8: Model for SECPT condition - moderation by cortisol level

**Models**	**AIC**	**BIC**
(1) Baseline Stress Model	6863	6921
(2) Cortisol Stress Model	6864	6928

Note: Note: There was no statistically significant difference in model fit.

Model specifications

Baseline Stress Model:

BID.c ~ 1 + RANGE.c + EV.c + (1 + RANGE.c + EV.c | ID)

Cortisol Stress Model:

BID.c ~ 1 + RANGE.c + EV.c + CORT_CHANGE + (1 + RANGE.c + EV.c | ID)

In this model comparison for the SECPT condition only, we compared the *Baseline Stress Model* (which was identical to the Baseline Model above), which had *Expected Value* and *Range* as fixed effects (and as additional random effects, to account for individual differences in these parameters, as well as *Participant* as a random effect) with the *Cortisol Stress Model*, which included participants' cortisol levels as an additional fixed effect. However, there was no improvement of model fit when cortisol level was included.

Questionnaire Results

Supplementary Table 9: Descriptive results questionnaires

Questionnaire Scale	Control Condition Mean (SD)	SEPCT Condition Mean (SD)	Welch's t-test
BFI Anxiety	11.76 (3.61)	12.82 (3.88)	t(64.89) = 1.16, p = .24, d = .28
BFI Volatility	9.79 (3.66)	9.68 (3.80)	t(64.99) = 0.12, p = .90, d = .03
BFAS Volatility	24.94 (7.32)	25.32 (8.39)	t(64.29) = 0.20, p = .84, d = .05
BFAS Withdrawal	26.97 (6.89)	28.85 (7.32)	t(64.94) = 1.08, p = .28, d = .26
5DCR Joyous Exploration	24.76 (5.27)	26.09 (5.11)	t(64.76) = 1.05, p = .30, d = .26
5DCR Deprivation Sensitivity	21.06 (5.71)	22.50 (5.87)	t(65.00) = 1.02, p = .31, d = .25
5DCR Stress Tolerance	21.64 (6.08)	22.88 (6.40)	t(64.97) = 0.82, p = .42, d = .20
5DCR Social Curiosity	25.94 (4.76)	25.85 (6.36)	t(61.06) = 0.06, p = .95, d = .02
5DCR Thrill Seeking	17.79 (6.64)	18.15 (6.12)	t(64.20) = 0.23, p = .82, d = .06
IUS Prospective	20.36 (4.43)	18.91 (4.71)	t(64.94) = 1.30, p = .20, d = .32
IUS Inhibitory	11.76 (3.94)	10.50 (3.96)	t(64.96) = 1.30, p = .20, d = .32
DOSPERT Ethical	15.49 (6.51)	15.68 (5.66)	t(63.20) = 0.13, p = .90, d = .03
DOSPERT Financial	17.42 (7.55)	17.97 (8.69)	t(64.22) = 0.28, p = .78, d = .07
DOSPERT Health Safety	16.27 (6.54)	17.47 (7.73)	t(63.82) = 0.69, p = .50, d = .17
DOSPERT Recreational	21.55 (8.99)	22.06 (8.63)	t(64.67) = 0.24, p = .81, d = .06
DOSPERT Social	28.91 (5.47)	31.24 (6.44)	t(63.89) = 1.59, p = .12, d = .39

Note: BFI = BFI-2 anxiety and emotional volatility questionnaire (Soto & John, 2017); BFAS = Big Five Aspects Scale (DeYoung et al., 2007); 5DCR = Five-Dimensional Curiosity Scale-Revised (Kashdan et al., 2020); IUS = Intolerance for Uncertainty Scale (Carleton et al., 2007); DOSPERT = Domain-specific Risk-taking Scale (Blais & Weber, 2006). No statistical differences were found between experimental groups on any scale of subscale.

Supplementary Table 10: Pearson Correlations between Questionnaires and Scales

**Scale**	(1)	(2)	(3)	(4)	(5)	(6)	(7)	(8)	(9)	(10)	(11)	(12)	(13)	(14)	(15)	(16)
(1) BFI Anxiety	-															
(2) BFI Volatility	.73*	-														
(3) BFAS Volatility	.69*	.78*	-													
(4) BFAS Withdrawal	.79*	.67*	.70*	-												
(5) 5DC Joyous Exploration	-.25*	-.25*	-.25*	-.44*	-											
(6) 5DC Deprivation Sensitivity	.15	.15	.13	.09	.32*	-										
(7) 5DC Stress Tolerance	-.52*	-.52*	-.53*	-.58*	.26*	-.19	-									
(8) 5DC Social Curiosity	-.01	-.01	-.04	.01	.10	.05	.07	-								
(9) 5DC Thrill Seeking	-.10	.05	.11	-.20	.47*	.02	.14	.15	-							
(10) IUS Prospective	.41*	.36*	.34*	.36*	-.21	.39*	-.61*	.04	-.23	-						
(11) IUS Inhibitory	.44*	.48*	.44*	.48*	-.27*	.26*	-.68*	-.09	-.11	.68*	-					
(12) DOSPERT Ethical	.05	.17	.18	.18	.02	.14	-.17	.23	.38*	.03	.10	-				
(13) DOSPERT Financial	-.17	-.14	-.01	-.11	.22	.28*	.05	.16	.40*	-.05	.02	.47*	-			
(14) DOSPERT Health/Safety	-.05	.07	.13	.08	.03	.12	-.02	.12	.44*	.01	.07	.67*	.35*	-		
(15) DOSPERT Recreational	-.09	-.11	-.01	-.08	.43*	.20	.13	.45*	.55*	-.10	-.01	.46*	.58*	.50*	-	
(16) DOSPERT Social	-.17	-.13	.03	-.09	.29*	-.01	.31*	.20	.42*	-.41*	-.45*	.36*	.36*	.46*	.40*	-

Note: * indicates $p < .05$

Supplementary Table 11: Correlations between questionnaire results and individuals' parameters of the *Baseline Model*

Questionnaire Scale	Intercept<br>*r* [CI]	Range<br>*r* [CI]	Expected Value<br>*r* [CI]
BFI Anxiety	.097 [-.146, .33]	.108 [-.136, .339]	-.168 [-.393, .075]
BFI Volatility	.06 [-.183, .296]	.066 [-.177, .301]	-.112 [-.343, .132]
BFAS Volatility	.055 [-.187, .292]	.153 [-.091, .379]	**-.255* [-.466, -.015]**
BFAS Withdrawal	-.004 [-.244, .236]	-.007 [-.247, .233]	-.184 [-.406, .059]
5DCR Joyous Exploration	.052 [-.191, .289]	-.123 [-.353, .12]	.043 [-.199, .281]
5DCR Deprivation Sensitivity	-.022 [-.261, .219]	**-.283* [-.49, -.045]**	.101 [-.143, .333]
5DCR Stress Tolerance	-.113 [-.344, .131]	-.08 [-.314, .163]	.089 [-.155, .322]
5DCR Social Curiosity	.101 [-.143, .333]	-.155 [-.381, .088]	.178 [-.065, .401]
5DCR Thrill Seeking	.021 [-.22, .26]	.146 [-.097, .373]	.087 [-.157, .32]
IUS Prospective	.093 [-.15, .326]	-.15 [-.376, .094]	-.001 [-.241, .239]
IUS Inhibitory	.091 [-.153, .324]	-.129 [-.358, .115]	-.036 [-.274, .206]
DOSPERT Ethical	.142 [-.102, .369]	-.125 [-.355, .119]	**.288* [.052, .494]**
DOSPERT Financial	.132 [-.112, .361]	.013 [-.228, .252]	.036 [-.206, .274]
DOSPERT Health Safety	.103 [-.141, .335]	-.077 [-.312, .166]	.187 [-.056, .409]
DOSPERT Recreational	.214 [-.027, .432]	-.056 [-.292, .187]	.207 [-.035, .426]
DOSPERT Social	.028 [-.214, .266]	.141 [-.103, .369]	-.016 [-.256, .225]

Note: Correlations are Pearson correlation coefficients; * indicates significance (without corrections for multiple comparisons) at $p < .05$. BFI = BFI-2 anxiety and emotional volatility questionnaire (Soto & John, 2017); BFAS = Big Five Aspects Scale (DeYoung et al., 2007); 5DCR = Five-Dimensional Curiosity Scale-Revised (Kashdan et al., 2020); IUS = Intolerance for Uncertainty Scale (Carleton et al., 2007); DOSPERT = Domain-specific Risk-taking Scale (Blais & Weber, 2006).

No significant correlations were found after correction for multiple comparisons. When no corrections were applied, the following correlations were significant. (Note that because of the explorative nature of these analyses and the relatively small sample, we refrain from interpreting these results, but simply report them here for completeness.)

The BFAS Volatility scale, a subscale of Neuroticism related to sensitivity to threat and punishment, and anger in particular (DeYoung et al., 2007), correlated negatively with the *Expected Value* parameter. This means that participants' bids for non-instrumental information were relatively less driven by the expected value of the lottery when they scored higher on this scale.

Deprivation Sensitivity scale of the 5DCR, which measures the anxiety and frustration experienced when people cannot access information desired (Kadshan et al., 2020), correlated negatively with the *Range* parameter. This means that participants'

bids for non-instrumental information were relatively less driven by the range between the available reward values when they scored higher on this scale.

Finally, The DOSPERT Ethical scale, which measures risk-taking in ethical decisions and behaviour (Blais & Weber, 2006), correlated positively with the *Expected Value* parameter. This means that participants' bids for non-instrumental information were relatively more driven by the expected value of the lottery when they scored higher on this scale.

Supplementary References

Blais A-R, Weber E (2006). A Domain-Specific Risk-Taking (DOSPERT) scale for adult populations. Judgement Dec Making, 1(1), 33-47.

DeYoung CG, Quilty LC, Peterson JB (2007). Between facets and domains: 10 aspects of the Big Five. J Pers Soc Psychol, 93(5), 880-896.

Kashdan TB, Disabato DJ, Goodman FR, McKnight PE. 2020. The Five-Dimensional Curiosity Scale Revised (5DCR): Briefer subscales while separating overt and covert social curiosity. Pers Individ Differ, 157, 109836.

Soto CJ, John OP (2017). The next Big Five Inventory (BFI-2): Developing and assessing a hierarchical model with 15 facets to enhance bandwidth, fidelity, and predictive power. J Pers Soc Psychol, 113(1), 117-143.
